# Supplementary material for: tRNA ligase structure reveals kinetic competition between non-conventional mRNA splicing and mRNA decay
Source: eLife. 2019 Jun 25;8:e44199. doi: 10.7554/eLife.44199 (PMC6592678; doi:10.7554/eLife.44199)
Supplement: Supplementary file 1. [file elife-44199-supp1.docx]

| Name | Description |
| --- | --- |
| pPW3206 | pET15b-His_6_-*ct*Trl1 [full length] |
| pPW3207 | pET15b-His_6_-*ct*Trl1-LIG |
| pPW3208 | pET15b-His_6_-*ct*Trl1-CPD |
| pPW3209 | pET47b-His_6_-*sc*Trl1-LIG-WT |
| pPW3210 | pET47b-His_6_-*sc*Trl1-LIG-H148Y |
| pPW3211 | p416ADH |
| pPW3212 | p416ADH-*sc*Trl1-H148Y-full |
| pPW3213 | p416ADH-*sc*Trl1-H148Y-LIG |
| pPW3214 | p416ADH-*ct*Trl1-H182Y-full |
| pPW3215 | p416ADH-*ct*Trl1-H182Y-LIG |
| pPW3420 | pET28a-His6-RtcA |
