# Supplementary material for: tRNA ligase structure reveals kinetic competition between non-conventional mRNA splicing and mRNA decay
Source: eLife. 2019 Jun 25;8:e44199. doi: 10.7554/eLife.44199 (PMC6592678; doi:10.7554/eLife.44199)
Supplement: Supplementary file 2. [file elife-44199-supp2.docx]

| **Strain**  **(alias)** | **Short description** | **Genotype** | **Plasmid** |
| --- | --- | --- | --- |
| yPW2089 (JPY001) | WT | *MATa leu2-3,112 TRP1 can1-100 ura3-1 ADE2 his3-11,15* | - |
| yPW2090 (JPY003) | *ski2Δ* | *MATa leu2-3,112 TRP1 can1-100 ura3-1 ADE2 his3-11,15 ski2Δ::hphNT1* | - |
| yPW2091 (JPY004) | *xrn1Δ* | *MATa leu2-3,112 TRP1 can1-100 ura3-1 ADE2 his3-11,15 xrn1Δ::hphNT1* | - |
| yPW2092 (JPY005) | *trl1-H148Y* | *MATa leu2-3,112 trp1-1 can1-100 ura3-1 ade2-1 his3-11,15 trl1-H148Y* | - |
| yPW2093 (JPY006) | *trl1-H148Y ski2Δ* | *MATa leu2-3,112 trp1-1 can1-100 ura3-1 ade2-1 his3-11,15 trl1-H148Y ski2Δ::hphNT1* | - |
| yPW2094 (JPY007) | *trl1-H148Y xrn1Δ* | *MATa leu2-3,112 trp1-1 can1-100 ura3-1 ade2-1 his3-11,15 trl1-H148Y xrn1Δ::hphNT1* | - |
| yPW2095 (JPY038) | WT  p416ADH | *MATa leu2-3,112 TRP1 can1-100 ura3-1 ADE2 his3-11,15* | pPW3211 |
| yPW2096 (JPY033) | *trl1-H148Y*  p416ADH | *MATa leu2-3,112 trp1-1 can1-100 ura3-1 ade2-1 his3-11,15 trl1-H148Y* | pPW3211 |
| yPW2097 (JPY034) | *trl1-H148Y*  p416ADH-*sc*Trl1-H148Y | *MATa leu2-3,112 trp1-1 can1-100 ura3-1 ade2-1 his3-11,15 trl1-H148Y* | pPW3212 |
| yPW2098 (JPY035) | *trl1-H148Y*  p416ADH-*sc*Trl1-H148Y-LIG | *MATa leu2-3,112 trp1-1 can1-100 ura3-1 ade2-1 his3-11,15 trl1-H148Y* | pPW3213 |
| yPW2099 (JPY036) | *trl1-H148Y*  p416ADH-*ct*Trl1-H182Y | *MATa leu2-3,112 trp1-1 can1-100 ura3-1 ade2-1 his3-11,15 trl1-H148Y* | pPW3214 |
| yPW2100 (JPY037) | *trl1-H148Y*  p416ADH-*ct*Trl1-H182Y-LIG | *MATa leu2-3,112 trp1-1 can1-100 ura3-1 ade2-1 his3-11,15 trl1-H148Y* | pPW3215 |
| yPW2101 (JPY020) | *trl1-H148Y dom34Δ* | *MATa leu2-3,112 trp1-1 can1-100 ura3-1 ade2-1 his3-11,15 trl1-H148Y dom34Δ::natNT2* | - |
| yPW2102 (JPY021) | *trl1-H148Y dom34Δ ski2Δ* | *MATa leu2-3,112 trp1-1 can1-100 ura3-1 ade2-1 his3-11,15 trl1-H148Y dom34Δ::natNT2 ski2Δ::hphNT1* | - |
| yPW2103 (JPY066) | WT 3xHA-*HAC1* | *MATa leu2-3,112 TRP1 can1-100 ura3-1 ADE2 his3-11,15* 3xHA-*HAC1* | - |
| yPW2104 (JPY067) | *ski2Δ *3xHA-*HAC1* | *MATa leu2-3,112 TRP1 can1-100 ura3-1 ADE2 his3-11,15* 3xHA-*HAC1 ski2Δ::hphNT1* | - |
| yPW2105 (JPY068) | *xrn1Δ *3xHA-*HAC1* | *MATa leu2-3,112 TRP1 can1-100 ura3-1 ADE2 his3-11,15* 3xHA-*HAC1 xrn1Δ::hphNT1* | - |
| yPW2106 (JPY088) | *dom34Δ ski2Δ *3xHA-*HAC1* | *MATa leu2-3,112 TRP1 can1-100 ura3-1 ADE2 his3-11,15* 3xHA-*HAC1* | - |
| yPW2107 (JPY069) | *trl1-H148Y* 3xHA-*HAC1* | *MATa leu2-3,112 trp1-1 can1-100 ura3-1 ade2-1 his3-11,15 trl1-H148Y* 3xHA-*HAC1* | - |
| yPW2108 (JPY070) | *trl1-H148Y ski2Δ *3xHA-*HAC1* | *MATa leu2-3,112 trp1-1 can1-100 ura3-1 ade2-1 his3-11,15 trl1-H148Y* 3xHA-*HAC1 ski2Δ::hphNT1* | - |
| yPW2109 (JPY087) | *trl1-H148Y xrn1Δ *3xHA-*HAC1* | *MATa leu2-3,112 trp1-1 can1-100 ura3-1 ade2-1 his3-11,15 trl1-H148Y* 3xHA-*HAC1 xrn1Δ::hphNT1* | - |
| yPW2110 (JPY072) | *trl1-H148Y dom34Δ ski2Δ* 3xHA-*HAC1* | *MATa leu2-3,112 trp1-1 can1-100 ura3-1 ade2-1 his3-11,15 trl1-H148Y* 3xHA-*HAC1 dom34Δ::natNT2 ski2Δ::hphNT1* | - |
| yPW2111 (JPY015) | *trl1-H148Y hac1Δ* | *MATa leu2-3,112 trp1-1 can1-100 ura3-1 ade2-1 his3-11,15 trl1-H148Y hac1Δ::natNT2* | - |
| yPW2112 (JPY016) | *trl1-H148Y hac1Δ ski2Δ* | *MATa leu2-3,112 trp1-1 can1-100 ura3-1 ade2-1 his3-11,15 trl1-H148Y hac1Δ::natNT2 ski2Δ::hphNT1* | - |
| yPW2113 (JPY017) | *trl1-H148Y hac1Δ xrn1Δ * | *MATa leu2-3,112 TRP1 can1-100 ura3-1 ADE2 his3-11,15* | - |
