# Supplementary material for: tRNA ligase structure reveals kinetic competition between non-conventional mRNA splicing and mRNA decay
Source: eLife. 2019 Jun 25;8:e44199. doi: 10.7554/eLife.44199 (PMC6592678; doi:10.7554/eLife.44199)
Supplement: Supplementary file 3. [file elife-44199-supp3.docx]

| **Gene** | **Forward primer** | **Reverse primer** | **Number of cycles** | **Length of amplicon [bp]** |
| --- | --- | --- | --- | --- |
| *HAC1*  (U and S) | CTGGCTGACCACGAAGACGC | CGATTGTCTTCATGAAGTGATGAAG | 23 | 723 (U)  471 (S) |
| *HAC1*  (5’ exon) | GTAATTCGCAATCGAACTTGGCTATCC | CGAGGCCACCGCATCAAAC | 23 | 545 |
| 18S rRNA (*RDN18-1*) | CTAACCTTGAGTCCTTGTGGCTC | CCTTATTGTGTCTGGACCTGG | 21 | 504 |
